# Could a defective epithelial sodium channel lead to bronchiectasis

**DOI:** 10.1186/1465-9921-9-46

**Published:** 2008-05-28

**Authors:** Isabelle Fajac, Marion Viel, Sébastien Sublemontier, Dominique Hubert, Thierry Bienvenu

**Affiliations:** 1 Service d'Explorations Fonctionnelles, AP-HP, Hôpital Cochin, Paris, France; 2 Université Paris Descartes, UPRES EA 2511, Faculté de Médecine, Paris, France; 3 Laboratoire de Biochimie et Génétique Moléculaires, AP-HP, Hôpital Cochin, Paris, France; 4 Service de Pneumologie, AP-HP, Hôpital Cochin, Paris, France; 5 Université Paris Descartes, Institut Cochin, CNRS (UMR 8104), Paris, France; 6 Inserm U567, Paris, France

## Abstract

**Background:**

Bronchiectasis is defined as a permanent dilation of the airways arising from chronic bronchial inflammation/infection. In 50% of cases, no etiology can be identified. Recently, the role of the epithelial sodium channel ENaC has been pointed out in the pathophysiology of cystic fibrosis, a disease due to mutations in the *CFTR *gene and causing bronchiectasis in the airways. Moreover, it was found that transgenic mice overexpressing *ENaCβ *present cystic fibrosis-like lung disease symptoms. Our aim was to evaluate if a defective ENaC protein could be involved in the development of bronchiectasis.

**Methods:**

We extensively analysed *ENaCβ *and *γ *genes in 55 patients with idiopathic bronchiectasis and without two mutations in the coding regions of *CFTR*. Thirty-eight patients presented functional abnormalities suggesting impaired sodium transport (abnormal sweat chloride concentration or nasal potential difference measurement), and 17 had no such evidence.

**Results:**

Sequencing of the exons and flanking introns of the *ENaCβ *and *γ *gene identified five different amino-acid changes (p.Ser82Cys, p.Pro369Thr, p.Asn288Ser in *ENaCβ *; and p.Gly183Ser, p.Glu197Lys in *ENaCγ*) in heterozygous state in 8 patients. The p.Ser82Cys amino-acid change was found in 3 unrelated patients who were also heterozygous for a *CFTR *mutation or variant (1 p.F508del, 1 IVS8-5T, and 1 IVS8-5T:1716G>A (p.E528E)). The other mutations were found in patients without *CFTR *mutation, the p.Glu197Lys mutation in 2 patients and the other variants in single patients. Among the 8 patients bearing an *ENaC *mutation, 5 had functional abnormalities suggesting impaired sodium transport.

**Conclusion:**

Our results suggest that several variants in *ENaCβ *and *γ *genes might be deleterious for ENaC function and lead to bronchiectasis, especially in patients who are trans-heterozygotes for *ENaCβ/CFTR *mutations or variants.

## Background

Bronchiectasis is an abnormal dilation of bronchi due to the destruction of their walls. The main symptoms associated with bronchiectasis are chronic cough, purulent sputum and recurrent lower respiratory tract infections, and most patients show a varying degree of airflow obstruction. In approximately 50% of cases, causative factors are identified such as childhood infection, immune defects, allergic bronchopulmonary aspergillosis, aspiration of irritants, cystic fibrosis, primary ciliary dyskinesia, rheumatoid arthritis, other connective tissue disorders, ulcerative colitis, and alpha-1 antitrypsin deficiency. The remaining 50% of cases without identified etiology are classified as idiopathic [1].

The amiloride-sensitive epithelial sodium channel (ENaC) allows passive transport of sodium into the cells. It is expressed in epithelial cells lining the renal tubule, the respiratory airways and alveoli, the distal colon and the duct of several exocrine glands, such as salivary and sweat glands. ENaC is composed of three subunits (alpha, beta and gamma) in the proposed stoichiometry 2α :1β :1γ, or 3α :3β :3γ [2,3]. Each ENaC subunit activity may be regulated by other proteins such as CFTR (Cystic Fibrosis Transmembrane conductance Regulator) which is the protein involved in cystic fibrosis [4]. Human clinical disorders due to *ENaC *mutations are a rare occurrence. However, they have been well described. Liddle's syndrome (OMIM 177200), an autosomal dominant form of volume-expanded low-renin hypertension is caused by gain-of-function mutations in the β or γ ENaC subunits [5]. In contrast, loss-of-function mutations in the α, β or γ ENaC subunits have been found in autosomal recessive pseudohypoaldosteronisme type I (PHA-I) (OMIM 264350) with salt wasting, hyperkalemia and metabolic acidosis [6]. In PHA-I patients, there is also a defective sodium transport in the sweat glands that causes elevated sweat chloride and sodium concentrations. Hence, as sweat testing is the standard diagnostic test for cystic fibrosis, PHA-I is a classic differential diagnosis of elevated sweat chloride concentration, although usually readily distinguishable from cystic fibrosis.

Recently, the ENaC channel has been shown to play a critical role in lung and airway mice physiology : ENaCα knock-out mice died shortly after birth of respiratory failure due to an inability to clear fluid from the alveolar space [7]. Moreover, mice overexpressing ENaCβ subunit, but not α or γ, developed cystic-fibrosis-like disease with mucus obstruction and poor bacterial clearance [8]. In humans, cystic fibrosis disease in the airways is linked to the combined defects of failure to secrete chloride and of accelerated sodium transport resulting from the absence of ENaC inhibition by the defective CFTR protein [9]. Moreover, some respiratory abnormalities have been reported in patients with clinical disorders due to ENaC mutations. Hence, in Liddle's syndrome, an increased sodium channel activity in the nasal epithelium was observed [10]. In PHA-I, patients frequently exhibit respiratory tract disease, especially up to the age of 5–6 years [11] and in some patients, a defect in nasal epithelial sodium transport has been described with an excess of airway surface liquid [12].

Recently, sequencing of the exons and flanking introns of the genes encoding the α, β, and γ subunits of ENaC in twenty non-classic CF patients, that is patients with bronchiectasis and elevated sweat chloride concentrations but without two *CFTR *mutations, identified in four patients five missense mutations (one in *ENaCα *and four in *ENaCβ*) and one splicing mutation in the 3' splice site of *ENaCβ *intron 12 [13]. Our aim was to evaluate if a defective ENaC protein could be involved in the development of bronchiectasis. To do so, we extensively analysed *ENaCβ *and *ENaCγ *genes in 55 patients with idiopathic bronchiectasis and identified 8 patients bearing one mutation in the *ENaCβ *or *γ *genes. One mutation was found in 3 unrelated patients who were also heterozygous for a *CFTR *mutation or variant. Moreover, 5 patients bearing one mutation in the *ENaCβ *or *γ *genes had functional abnormalities in the nasal epithelium or in the sweat glands suggesting impaired sodium transport.

## Material and Patients

### Patients and controls

We studied 55 patients (13 men and 42 women) with diffuse idiopathic bronchiectasis recruited from the respiratory physiology department of Cochin University Hospital, Paris, France. Bronchiectasis was diagnosed on chronic cough with purulent sputum and bronchial dilation involving more than one lobe on high-resolution computed tomography scanning [14]. A complete genomic screening for *CFTR *gene mutations by denaturing high performance liquid chromatography (DHPLC) and direct sequencing analysis was performed and no patient carried two pathogenic *CFTR *mutations. No other known etiology of bronchiectasis was found such as infectious causes, immune disorders, primary ciliary dyskinesia or chronic rheumatic inflammatory conditions as assessed by : complete medical history, blood investigations (serum protein electrophoresis, immunoglobulins (Ig), IgG subclasses, *Aspergillus fumigatus *radioallergosorbent (RAST), autoantibodies including rheumatoid factor, α1-antitrypsin) and nasal mucociliary clearance (measured by the saccharin test and nasal nitric oxide measurement). No patient had a history of salt wasting or a familial history of respiratory or metabolic diseases, and all patients were normotensive.

Sweat test and nasal PD measurements were performed on the 55 patients in order to study sodium transport in the sweat glands and in the nasal epithelium. Nasal PD measurements were performed by using 2 silver/silver chloride electrodes connected to a high impedance voltmeter, one electrode being in contact with the inferior surface of the nasal inferior turbinate and the other, with the subcutaneous tissue of the forearm [15]. The basal nasal PD reflects the basal rate of nasal sodium absorption [15]. Group 1 (38 patients) was defined as patients who had either abnormal sweat chloride concentration (13 patients) of more than 40 mmol per liter in a sample of at least 75 mg of sweat induced by means of pilocarpine iontophoresis [16] or abnormal basal nasal PD (> |-30| mV in 14 patients or < |-10| mV in 11 patients) measured as described by Knowles et al. [15]. When the basal nasal PD was above |-30| mV, a pharmacological study was performed as described by Knowles et al. [15]. This pharmacological study allows to explore each component of nasal epithelial ion transport by using various pharmacological agents such as amiloride, a blocker of epithelial sodium channel, a low chloride solution which allows the determination of basal chloride conductance and isoproterenol which explores CFTR-related chloride transport [15]. Group 2 (17 patients) included patients with no evidence of impaired sodium transport as evaluated by normal sweat chloride concentrations (< 40 mmol/L) and normal basal PD (≥ |-10| and ≤ |-30| mV). Values of forced expiratory volume in 1 second (FEV1) and forced vital capacity (FVC) were determined on the day of nasal PD measurements and expressed as percentages of predicted values.

A control group comprising 50 subjects of Caucasian origin with no pulmonary disease were studied for the *CFTR *and *ENaC *genes. All individuals gave written informed consent.

### Methods

Genomic DNA was extracted from peripheral blood lymphocytes according to standard protocols and was used to amplify the thirteen exons (twelve coding exons; exon 2 to exon 13) and the flanking sequences of the *ENaCβ *and *ENaCγ *genes. Because lung-specific overexpression of ENaCα was not associated with raised Na^+ ^transport rates or lung disease in mouse [8], we did not study this subunit. All samples were collected after a written informed consent was obtained.

**Figure 1:**
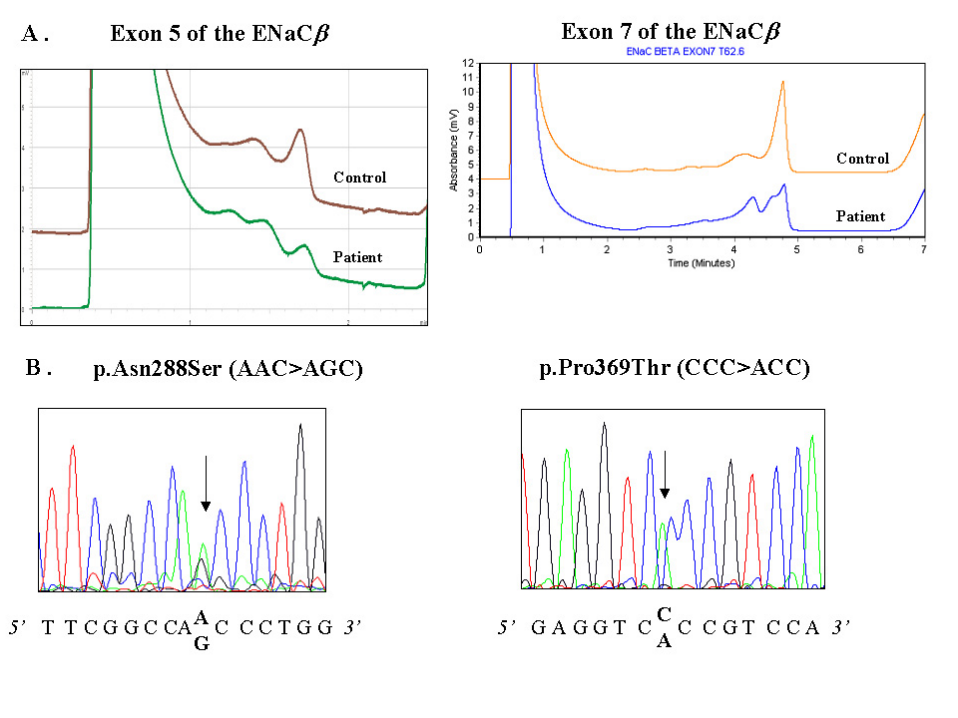
(A) Denaturing high performance liquid chromatography patterns of polymerase chain reaction products corresponding to exon 5 and 7 of *ENaCβ* : DHPLC profile corresponding to normal exon sequence (control), and altered DHPLC because of a substitution. (B) Fluorescence sequence analysis of exon 5 and exon 7 of the *ENaCβ *gene, using the forward primer. Arrows indicate position of the p.Asn288Ser and p.Pro369Thr mutations in exon 5 and 7, respectively.

The human *ENaCβ *and *ENaCγ *genes are located on chromosome 16, composed of thirteen exons and encode a 640 and 649 aminoacid protein, respectively. The ATG start codon is located in exon 2 and the TGA stop codon is located in exon 13. The coding regions of *ENaCβ and ENaCγ *have been amplified in twelve fragments. Primer sequences have been previously described [17]. Reactions were performed in a volume of 50 μl containing 50 mM Tris-HCl (pH 8.4), 1.5 mM MgCL_2_, 200 μM of all four deoxynucleotides, 0.5 mM each of the primers, 2.5 units of Taq Polymerase (Gold, Perkin Elmer) and 100 ng template DNA. Forty cycles were then performed with denaturation for 30 sec at 72°C. The DNA synthesis step of the final cycle was extended to 7 min. For DHPLC analysis, the amplified DNA was heated at 94°C for 7 min, and at 55°C for 4 min to favour formation of heteroduplexes. Mutation analysis of the patients with idiopathic bronchiectasis was performed by using DHPLC (Wave DNA fragment analysis system, DNAsep column; Transgenomics). DHPLC conditions were chosen according to the Wavemaker program (Transgenomics, Santa Clara, CA, USA) as previously described [17]. PCR products were subjected to chromatography using appropriate temperatures and acetonitrile gradient at a flow rate of 0.9 ml/mn, and those showing an abnormal DHPLC profile were directly sequenced on an automated sequencer (ABI 3100, Applied Biosystems, USA) using the BigDye Terminator method (Figure 1).

The influence of base substitutions on putative exonic splice element (ESE) sites was determined with the ESEfinder program [18].

### Statistics

The Hardy-Weinberg equation tested allelic disequilibrium for the common polymorphisms. Comparison between subjects grouped on the basis of their phenotypes was accomplished by Fisher's exact test (two tailed) for categorical variables and by Mann-Whitney U test for continuous variables. P values below 0.05 were defined as significant.

## Results

### Patients

All patients were caucasian europeans except for 4 patients who originated from Africa (2 patients), Asia (1 patient) and South America (1 patient). Table 1 shows the clinical characteristics of the patients. In both groups, some patients carried one *CFTR *mutation or variant : in Group 1, 5 patients were heterozygous for the p.F508del, 1 for the 2183AA>G mutation, 1 for the 4375-20G>A variant, 2 for the IVS8-5T allele and 3 for the c.1716G>A (p.E528E) variant. In Group 2, 2 patients were heterozygous for the p.F508del, 2 for the p.R117H-7T mutation, 3 for the IVS8-5T allele, and 1 for the c.1717G>A (p.E528E) variant. There was no statistical difference in the frequencies of *CFTR *mutations between the 2 groups.

**Table 1:** Main characteristics of the 55 patients with idiopathic bonchiectasis

Variable	Group 1 (n = 38)	Group 2 (n = 17)	P value
Age (years)	54 ± 3	52 ± 4	NS
BMI (Kg.m^-2^)	23 ± 1	20 ± 1	NS
FEV1 (% predicted)	86 ± 4	75 ± 6	NS
Sweat chloride (mmol/L)	34 ± 3	22 ± 2	0.03
Basal nasal PD (mV)	-23 ± 2	-18 ± 1	NS

Results are expressed as mean ± SEM; BMI : body mass index, FEV1 : forced expiratory volume in 1 second, PD : potential difference.

### Screening of the *ENaC *gene

All the exons and exon-intron junctions of the *ENaCβ *and *ENaCγ *gene were screened for mutations and/or polymorphisms by DHPLC in the 55 patients with bronchiectasis of unknown origin. DHPLC analysis revealed the absence of mutations or polymorphisms in exons 3, 4, 6, 9, 10, 11, 12 of the *ENaCβ *gene and in exons 2, 5, 6, 7, 8 and 12 of the *ENaCγ *gene.

### Mutations in the *ENaCβ *gene

Screening of the *ENaCβ *gene by DHPLC and direct sequencing revealed three different amino-acid changes : p.Ser82Cys c.245C>G in exon 2, p.Asn288Ser c.863A>G in exon 5 and p.Pro369Thr c.1105C>A in exon 7 (Figure 1). The p.Ser82Cys amino-acid change has been previously described [13] and was found in 3 unrelated patients. The two other mutations have not been previously described and were found each in one patient. The amino acids Ser82 and Asn288 are completely conserved in paralogues in human, canis, rabbit, rat and mouse. However, the amino acid Pro369 is not completely conserved in paralogues (Ala in mouse and rat) [19].

### Characteristics of the patients bearing mutations in the *ENaCβ *gene

These three missense mutations were identified 4 times in patients from Group 1 (2 : p.Ser82Cys, 1 : p.Pro369Thr and 1 : p.Asn288Ser; 4/38; 10.5%) and only once in patients from Group 2 (p.Ser82Cys; 1/17; 5.9%). None of these mutations were found in the ethnically-matched control group. Interestingly, the 3 patients bearing the p.Ser82Cys mutation were also heterozygous for a *CFTR *mutation or variant (1 p.F508del, 1 IVS8-5T, and 1 IVS8-5T:1716G>A (p.E528E)).

The main characteristics of the patients bearing a missense mutation in *ENaCβ *gene are shown in Table 2. All were caucasian europeans, except a 35-year old female who originated from South America. Most of them were quite elderly adults (≥ 60 years-old), all of them had a normal or subnormal respiratory function. None had a bronchial colonization by *Pseudomonas aeruginosa*. In patients from group 1, all but one had a normal or a very low basal nasal PD and a pharmacological study was not performed. The only patient with a high basal nasal PD in absolute value (- 43 mV) bore the mutation p.Pro369Thr. In this patient, the sweat chloride concentration was normal (22 mmol/L) and the PD response to pharmacological solutions was normal : it was reduced to – 22 mV with amiloride, a sodium channel inhibitor. A normal response was observed after perfusion of a low chloride solution (- 44 mV) and subsequently after perfusion of the β-agonist isoproterenol which induces cAMP dependent chloride conductance (- 56 mV), showing a functional CFTR protein.

**Table 2:** Main characteristics of the 5 patients with idiopathic bonchiectasis bearing a missense mutation in *ENaCβ *gene

	Age (years)	Sex	BMI (Kg.m^-2^)	FEV1 (%pred.)	*CFTR *mutation	*ENaCβ *mutation	Sweat Cl^- ^(mmol/L)	Basal PD (mV)
Group 1	66	F	22	77	IVS8-5T	p.Ser82Cys	44	- 13
	62	F	19	89	F508del	p.Ser82Cys	38	- 8
	35	F	23	86	none	p.Pro369Thr	22	- 43
	60	F	21	93	none	p.Asn288Ser	57	- 10
Group 2	67	M	20	80	IVS8-5T	p.Ser82Cys	28	- 22

F : female, M : male, BMI : body mass index, FEV1 : forced expiratory volume in 1 second, pred : predicted, PD : nasal potential difference.

### Polymorphisms in the *ENaCβ *gene

Four silent polymorphisms (p.Pro93Pro c.279C>T in exon 2; p.Phe293Phe c.879C>T in exon 5; p.Pro407Pro in exon 8; and p.Asn629Asn in exon 13) and one nucleotide change in the noncoding region (c.IVS12-17C>T) were also found. The silent polymorphism p.Phe293Phe was observed in six unrelated patients (6/55; 11%) (6 in group 1 (15%) vs 0 in Group 2 (0%)) and the common polymorphism p.Pro93Pro was identified at the heterozygous state in 18 patients (18/55; 33%) (13 in Group 1 and 5 in Group 2) and at the homozygous state in 9 patients (9/55; 16%) (5 in Group 1 (13%) and 4 in Group 2 (23%)). The two other silent polymorphisms (p.Pro407Pro and p.Asn629Asn) were only observed once.

### Mutations in the *ENaCγ *gene

Screening of the *ENaCγ*gene by DHPLC and direct sequencing revealed two amino-acid changes (p.Gly183Ser c.547G>A in exon 3 and p.Glu197Lys c.591G>A in exon 3).

### Characteristics of the patients bearing mutations in the *ENaCγ *gene

The p.Gly183Ser amino-acid change was found in a female patient from Group 1 who originated from Africa and the p.Glu197Lys amino-acid change was found in two unrelated patients from Group 2. None of these mutations were found in the ethnically-matched control group.

The main characteristics of the patients bearing a missense mutation in *ENaCγ*gene are shown in Table 3. None carried a *CFTR *mutation. The patient from Group 1 carrying the p.Gly183Ser mutation had normal sweat chloride concentrations (7 mmol/L), an abnormal basal nasal PD (- 39 mV) with normal response to amiloride (- 29 mV) and to low chloride solution (- 40 mV). No response was observed after perfusion with isoprenaline (- 40 mV). In Group 2, the 2 patients carrying the p.Glu197Lys were among the most severely affected patients of Group 2 with airway obstruction on pulmonary function tests.

**Table 3:** Main characteristics of the 3 patients with idiopathic bronchiectasis bearing a missense mutation in *ENaCγ *gene

	Age (years)	Sex	BMI (Kg.m^-2^)	FEV1 (%pred.)	*CFTR *mutation	*ENaCγ *mutation	Sweat Cl(mmol/L)	Basal PD (mV)
Group 1	36	F	20	90	none	p.Gly183Ser	7	- 39
Group 2	24	F	19	64	none	p.Glu197Lys	11	- 17
	46	M	22	35	none	p.Glu197Lys	17	- 18

F : female, M : male, BMI : body mass index, FEV1 : forced expiratory volume in 1 second, pred : predicted, PD : nasal potential difference.

### Polymorphisms in the *ENaCγ *gene

Six silent polymorphisms (p.Tyr129Tyr c.387T>C in exon 3; p.Ser145Ser c.435C>T in exon 3; p.Ileu158Ileu c.474T>C in exon 3; p.Gly183Gly c.549C>T in exon 3; p.Ser212Ser c.636C>T in exon 4; p.Val492Val c.1476A>G in exon 12; p.Leu649Leu c.1947 C>G in exon 13) and five intronic sequence variations (c.IVS7+14A>G; IVS7+69A>G; c.1371+29T>C; c.1432-7G>A; c.1432-106A>G) were also found. The common polymorphisms p.Tyr129Tyr and p.Ileu158Ileu (also named rs4365290 and rs5735, respectively) were observed in 28 patients (28/55 i.e. 51%; 18 (47%) in Group 1 and 10 (59%) in Group 2) at the heterozygous state. In the ensembl database which provides accurate analysis of the current human genome data [20], these variants were observed at the heterozygous state in 54%. The common polymorphism p.Leu649Leu (also named rs5723) was identified in 18 patients (18/55 i.e. 33%; 12 (32%) in Group 1 and 6 (35%) in Group 2) and was in perfect linkage with the two intronic sequence variations c.1371+29T>C and c.1432-7G>A. The common polymorphism p.Ser212Ser (also named rs16977041) was only observed in two unrelated patients from Group 1 (2/55; 4%) and the two other silent mutations p.Ser145Ser and p.Val492Val were only observed once (both from Group 1; 2%). None of the intronic nucleotide changes altered the consensus splice sites, suggesting that they are common polymorphisms rather than disease-causing mutations.

## Discussion

Our study shows that, out of 55 patients with bronchiectasis of unknown origin, 8 patients carried at least one missense mutation in *ENaCβ *or *ENaCγ *genes. Interestingly, the p.Ser82Pro mutation was found in 3 patients heterozygous for a *CFTR *mutation or variant. Functional tests to investigate the rate of sodium transport in the sweat glands or in the nasal epithelium were not contributive in suspecting the presence of these mutations since 3 patients out of the 8 patients bearing one *ENaC *mutation had a normal sweat test and a normal basal PD. However, in the 5 patients with abnormal functional tests bearing one *ENaC *mutation, the effect of the mutation on ENaC function might be hypothesized.

Liddle's syndrome is caused by nonsense, and frameshift mutations resulting mostly in truncation of carboxyl termini of β and γsubunits, and by missense mutations primarily located in a conserved PY motif (PPPXYXXL motif, codon 611 to 623). This leads to defective regulation of ENaC expression and activity, and gain-of-function [5,21]. PHA-I is mostly caused by nonsense, splice and frameshift mutations in the *ENaCα, β *and *γ*genes which produce truncated nonfunctional proteins, or missense mutations in the *ENaCα *and *β *genes that decrease ENaC trafficking to the cell surface. All these mutations cause loss-of-function of the ENaC protein and were reported in patients with a severe phenotype of PHA-I [22]. In contrast, out of 22 mutations causing PHA-I, only 3 were missense mutations and they were reported in PHA-I patients with a mild form of the disease [22]. While the non-missense mutations lead to absence of normal-length protein, missense mutations allow the synthesis of a normal-length subunit that is more likely to support a residual channel activity. In this study, 55 patients with bronchiectasis of unknown origin have been examined for mutations in coding regions of the *ENaCβ *and *γ *genes and 5 different missense mutations were identified. It can by hypothesized that the missense mutation resulting from the nucleotide substitution allowed normal sodium reabsorption in the kydneys (therefore avoiding the syndromes of pseudohypoaldosteronism or pseudoaldosteronism seen in PHA-I and Liddle's syndrome); but this missense mutation might mildly affect ENaC function in the airway epithelium, and this abnormal ENaC protein would modify the extent of sodium absorption and lead to airway disease and bronchiectasis.

Four of the missense mutations were detected in patients with functional abnormalities suggesting impaired sodium transport in the sweat glands or in the nasal epithelium : two variants (p.Pro369Thr, p.Asn288Ser in *ENaCβ) *were detected in this study for the first time and the other two (p.Ser82Cys and p.Gly183Ser) have been described before [13]. These variants have not been previously found in normal subjects in several studies [22-24] and were not detected in our ethnically-matched control group and in a "control" population of 56 cystic fibrosis patients with two pathogenic *CFTR *mutations [17]. They are therefore unlikely to be common polymorphisms. These variants were each found at the heterozygous state and the deleterious effect of this finding might be questioned. *In vitro *studies in the Xenopus laevis oocyte expression system could be performed to evaluate the precise effect of the new mutations we have found on ENaC structure and function. However, we did not performe these studies because it is thought that this system is not sensitive enough to detect little changes in ENaC activity and that only mutations leading to large changes in ENaC activity are liable to be detected [25]. Moreover, it is not known whether amphibian cells such as Xenopus oocytes possess the whole cellular machinery involved in the complex regulation of the ENaC in mammalian cells [26]. However, the functional abnormalities found in the patients speak for a role of the *ENaC *mutation in the pathophysiology of their airway disease. Hence, the patients bearing the p.Pro369Thr and p.Gly183Ser mutations had normal sweat chloride concentration (22 and 7 mmol/L, respectively) and a high basal PD (- 43 and - 39 mV, respectively) showing elevated nasal sodium transport. This pattern is similar to that described in Liddle's syndrome [10]. Indeed, in the presence of *ENaC *gain-of-function mutations such as in Liddle's syndrome, the sodium and chloride reabsorption in the sweat duct are normal leading to normal sweat chloride concentrations. In contrast, there is an increased sodium reabsorption in the airway epithelium causing an increased nasal PD. Therefore, the functional pattern we observed for sodium transport in the sweat gland and in the airway epithelium suggests that p.Pro369Thr and p.Gly183Ser are mutations causing a gain of ENaC function. As for the patient bearing the p.Asn288Ser mutation, she had high sweat chloride concentration (57 mmol/L) with low basal nasal PD (- 10 mV), displaying the pattern described for PHA-I patients [12]. In loss-of-function mutations in the *ENaC *gene as seen in PHA patients, the defective ENaC causes defective sodium and chloride reabsorption in the sweat duct and elevated sweat chloride and sodium concentrations. In the airways, the low sodium reabsorption leads to a low basal nasal PD. Therefore, the functional pattern we observed for the p.Asn288Ser mutation suggests it to be a loss-of-function mutation.

The p.Ser82Cys mutation was relatively common in our population (3/55; 5.4%) and was found in both groups, with or without functional abnormalities suggesting impaired sodium transport. The two patients from Group 1 bearing the p.Ser82Cys mutation had rather high sweat chloride concentrations (44 and 38 mmol/L) with rather low basal nasal PD (- 13 and - 8 mV), displaying the pattern described for PHA-I patients [12], that is patients bearing *ENaC *mutations causing a loss of function in the ENaC channel. However, this p.Ser82Cys mutation has been reported at a frequency of 2% in control subjects, thus making its mere presence deleterious doubtful [27]. Interestingly, the three patients bearing this variant also bore one *CFTR *mutation (p.F508del in one case) or one *CFTR *variant (IVS8-5T in one patient, and IVS8-5T-1716G>A in the other), which was not the case for the four patients bearing one other *ENaC *mutation. This p.Ser82Cys variant was not found in our cohort of 56 cystic fibrosis patients bearing two pathogenic *CFTR *mutations [17]. To date, a combination of one *ENaC *mutation, including this p.Ser82Cys mutation, plus a *CFTR *mutation, has been occasionally reported in patients with idiopathic bronchiectasis [27]. Here, we have identified three patients with bronchiectasis who are trans-heterozygotes for *ENaCβ/CFTR *mutations or variants, and these cases strongly suggest an interaction between different susceptibility factors in the pathogenesis of their airway disease. As it has been previously suggested in sporadic cases of PHA-I with various polymorphisms identified in the *ENaC *gene [21], this sporadic presentation of bronchiectasis with trans-heterozygotie for *ENaCβ/CFTR *mutations could be the result of digenic or multigenic expression and complex hereditary transmission.

Among the 8 patients bearing one missense mutation in *ENaCβ γ *gene, 3 had functional tests suggesting normal sodium transport in the sweat glands and in the nasal epithelium. This, together with the heterozygous state of the *ENaC *mutation, may suggest that this mutation does not play a role in the pathophysiology of these patients' airway disease. However, as one of the patient bore also one *CFTR *variant (IVS8-5T) and as the other two bore the same *ENaCγ *mutation (p.Glu197Lys) and were among the most affected patients of the group, the implication of the *ENaC *mutation can not be ruled out. If the last hypothesis is true, our results indicate that the functional tests studying sodium transport, such as sweat testing and nasal PD, are not always contributive in suspecting a mutation in the *ENaC *gene. Moreover, it strengthens our hypothesis that airway disease related to partly defective ENaC protein might be the result of complex susceptibility factors and this is all the more emphasized by the growing knowledge of all the accessory factors regulating ENaC [28].

In a group of 55 patients with idiopathic bronchiectasis, we identified 8 patients bearing one mutation in the *ENaCβ *or *γ *genes. Although the significance of these findings at the functional level requires further investigation, a defective ENaC protein may be involved in some patients with idiopathic bronchiectasis or cystic fibrosis-like lung disease with only one *CFTR *mutation identified.

## Competing interests

The authors declare that they have no competing interests.

## Authors' contributions

IF, DH and TB designed the study, MV and SS performed the genetic analysis, IF performed the nasal PD measurements. All authors have participated in the analyses of data. IF and TB had the major responsibility for drafting the manuscript. All authors read and approved the final manuscript.

## Aknowledgements

The authors are indebted to Prof. M. Aubier (Bichat Hospital, Paris), Dr L. Bassinet (Centre Hospitalier Intercommunal de Créteil), Dr P.R. Burgel (Cochin Hospital, Paris), Prof J. Cadranel (Tenon Hospital, Paris), Prof. L.J. Couderc (Foch Hospital, Suresnes), Prof. B. Crestani (Bichat Hospital, Paris), Dr N. Dufeu (Cochin Hospital, Paris), Prof. D. Dusser (Cochin Hospital, Paris), Dr I. Honoré (Centre Hospitalier Intercommunal de Créteil), Dr J. Lacronique (Cochin Hospital, Paris), Dr E. Rivaud (Foch Hospital, Suresnes) for referring the patients. This work was supported by a grant from the association Vaincre la Mucoviscidose.
